# A Lyophilizable Nanoparticle Vaccine Specific for a Novel Linear Neutralizing Epitope in the α2-α3 Helices of Domain 3 of Lethal Factor from *Bacillus anthracis*

**DOI:** 10.3390/toxins17080422

**Published:** 2025-08-20

**Authors:** Jon Oscherwitz, Kemp Cease, David Milich, Thomas Braun, Fen Yu, David Whitacre

**Affiliations:** 1 Division of Hematology-Oncology, Department of Internal Medicine, University of Michigan Medical School, Ann Arbor, MI 48105, USAfenyu@umich.edu (F.Y.); 2 Veterans Administration Ann Arbor Healthcare System, 2215 Fuller Road, Ann Arbor, MI 48105, USA; 3 VLP Biotech, Inc., 3030 Bunker Hill St., Ste 117D, San Diego, CA 92109, USA; dmilich@vlp-biotech.com (D.M.); dwhitacre@vlp-biotech.com (D.W.); 4 Department of Biostatistics, University of Michigan, Ann Arbor, MI 48105, USA; tombraun@umich.edu

**Keywords:** anthrax, virus-like particle, antibody, neutralization, lyophilization, lethal factor, *Bacillus anthracis*, vaccine

## Abstract

Anthrax remains a serious bioterrorism threat for which new and thermostable vaccines are needed. We previously demonstrated that immunization of rabbits with multiple-antigenic-peptide (MAP) vaccines elicit antibody (Ab) against the loop-neutralizing-determinant (LND), a cryptic linear neutralizing epitope in the 2β2-2β3 loop of protective antigen (PA) from *Bacillus anthracis* (*B. anthracis*), which mediates the complete protection of rabbits from inhalation spore challenge with *B. anthracis* Ames strain. Importantly, LND-specific Ab is not significantly elicited with PA-based vaccines. In the current study, we sought to identify a second unique neutralizing epitope which would also not overlap with the neutralizing specificities elicited by PA-based vaccines, and which could be combined with an LND vaccine as a prototype bivalent vaccine for anthrax. We evaluated linear peptide sequences in the α2-α3 helices of domain 3 of lethal factor (LF) in the form of virus-like particle (VLP) vaccines. Immunogenicity studies confirmed the presence of a 20-mer peptide sequence that is capable of eliciting protective levels of neutralizing Ab following two immunizations of rabbits using human-use adjuvants, and lyophilization of the VLPs did not diminish their immunogenicity. To our knowledge, this is the first demonstration that immunization with linear peptide sequences from LF can elicit protective levels of neutralizing Ab in vivo.

## 1. Introduction

There continues to be efforts to evaluate new, optimized, and stable vaccines for anthrax, which, owing to the relative simplicity of producing highly lethal weapons-grade spores from the bacterium *Bacillus anthracis* (*B. anthracis*), continues to represent a serious bioterrorist threat [1,2,3]. The currently licensed anthrax vaccine in the U.S., Biothrax^®^, is a culture filtrate from a toxigenic, non-encapsulated strain of *B. anthracis*, adsorbed to an Alum adjuvant, and is administered as five immunizations over 18 months with a requirement for yearly boosting to maintain antibody (Ab) titers [4]. Protective antigen (PA), the cell-binding component of the tripartite protein complex which includes lethal factor (LF) and edema factor, is the major immunogenic component of the vaccine and immunization with BioThrax^®^ elicits neutralizing Ab against PA which mediates protection in experimental animal models of anthrax inhalation spore challenge [5,6]. This protection is highly correlated with the ability of PA-specific Abs to neutralize lethal toxin (LeTx) in the toxin neutralization assay (TNA) [6,7,8,9,10,11,12].

Despite the established protective efficacy of Biothrax^®^ in experimental models of inhalation anthrax, including rabbits and non-human primates, the two commonly-accepted animal models for testing anthrax vaccines, efforts continue towards the development of new and more stable vaccines [2,13,14,15,16]. These efforts have been undertaken for several reasons. First, despite streamlining the immunization protocol to five immunizations over 18 months with yearly booster immunizations, it still remains a rigorous immunization protocol which reduces compliance [17]. Second, immunization is often accompanied by significant reactogenicity including local erythema, swelling, and pain, and is not infrequently associated with systemic symptoms including fatigue, myalgias and generalized discomfort [2,18,19]. Additionally, Biothrax^®^, as well as the licensed vaccine in the U.K, anthrax vaccine precipitated (AVP), both require a cold-chain. For Biothrax^®^, this necessitates the costly replacement of existing vaccine stocks in the U.S. strategic national stockpile at clearly defined intervals owing to chemical alterations in PA during storage which reduces the immunogenicity of the vaccine [20,21,22,23]. Finally, studies have shown that a proportion of Biothrax vaccinees (formerly AVA) develop comparatively low levels of neutralizing Ab. This could potentially leave vaccinees vulnerable in the event of an anthrax spore exposure [24].

We previously demonstrated that an epitope in the 2β2-2β3 loop of PA, referred to as the loop-neutralizing determinant (LND), represented a potent neutralizing epitope, and antibody elicited to this sequence displayed on MAPs or in a recombinant protein could elicit neutralizing Ab that is capable of mediating protective efficacy in rabbits from aerosol spore challenge with *B. anthracis* Ames strain [25,26,27]. The LND is found within a segment of PA that is critical to the translocation of edema and lethal factor into cells to form edema toxin and LeTx, respectively, and contains the chymotrypsin cleavage site [28,29,30]. Mutagenesis of the sequence within the LND binding motif abrogates the toxicity of LeTx, thereby rendering this neutralizing epitope comparatively resistant to the potential malicious re-engineering of PA in a manner meant to circumvent the effectiveness of neutralizing antibody [31,32,33]. Data has shown that Ab to the LND is not meaningfully elicited in rabbits, NHPs, and humans through immunization with PA-containing vaccines [25,34,35]. An LND vaccine, therefore, elicits a neutralizing specificity which does not overlap with the specificities currently elicited with PA-based anthrax vaccines and may represent a candidate for development into a stand-alone pre- or post-exposure vaccine for anthrax, or as an adjunct to current PA-based vaccines, including Biothrax^®^. Though the development of an LND-based vaccine holds promise, we sought to develop a bivalent epitope-specific vaccine which would obviate a single-point-of-failure associated with a vaccine containing only a single neutralizing epitope such as the LND. Additionally, a bivalent vaccine, containing two distinct neutralizing epitopes, would theoretically be expected to reach protective levels of neutralizing Ab titers more quickly than a vaccine targeting only the LND. We therefore endeavored in the current study to identify a linear neutralizing epitope in LF, which, like immunization with vaccines displaying the LND, would elicit neutralizing Ab specificities non-overlapping with the Ab specificities elicited by PA-only-containing vaccines, including Biothrax^®^. Towards these ends, we molecularly constructed virus-like particle (VLP) vaccines using the Woodchuck hepatitis core antigen capsid (WHcAg) displaying putative linear neutralizing epitopes from the α2-α3 helices of domain 3 of *B. anthracis* LF, and evaluated these prototype vaccines in rabbits. These VLPs are highly immunogenic, self-assembling nanoparticles comprising 240 capsid protein monomers, each displaying unique genetically-fused linear epitopes on the surface of the nanoparticle. VLPs are non-infectious, and unlike current PA-based vaccines like Biothrax^®^, the VLP vaccines are capable of being lyophilized for storage, but once reconstituted, maintain their original immunogenic profile [36,37]. The results of our studies show that a distinct linear neutralizing epitope exists within the α2-α3 helices of domain 3 of *B. anthracis* lethal factor. We demonstrate, for the first time, that the immunization of rabbits with a VLP displaying a linear peptidic neutralizing epitope from LF elicits protective levels of LF-specific neutralizing antibody in rabbits using human-use adjuvants.

## 2. Results

### 2.1. Molecular Construction and Immunogenicity of VLPs Displaying LF Peptide Sequences

**Figure 1:**
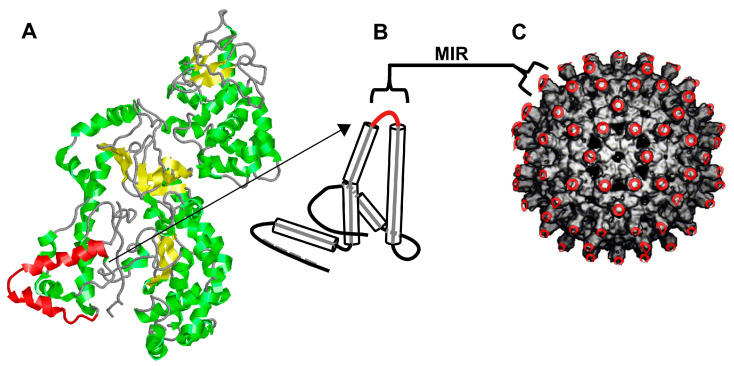
Molecular construction of the LF-VLPs. Shown in (**A**) is a protein structural model of LF based on the 1JKY crystal structure with the location of the VLP147 sequence (PDB a.a. 332–366) highlighted in red. In (**B**) is a diagrammatic representation of the protein structural model of the WHcAg monomer with the alpha-helices depicted as cylinders and the insertion site for the LF epitopes within the Major Immunogenic Region (MIR) of the WHcAg shown in red. Each WHcAg monomer forms dimers which then self-assemble into the nanoparticle (**C**) comprising 240 monomers, each displaying the MIR sequences (location depicted in red in the nanoparticle). Image in panel A was created in RasMol based on the 1JKY crystal structure of LF.

Several lines of evidence suggested that sequences found with the α2-α3 helices of domain 3 of LF may contain linear neutralizing epitopes. First, crystal structure analysis suggested that the linear sequences around a.a. 332–366 (PDB) were highly mobile and did not have a consistently defined structure. This is evident in the lack of resolution of these sequences in the crystal structures, for example, in PDB 1J7N, 1PWP, 1PWQ, and 1PWV, while the sequences were resolved when LF was bound to the N-terminal sequence of MAPKK2 in PDB 1JKY, likely through the stabilization of sequences in this region (Figure 1A). A somewhat similar pattern was also seen in our earlier work identifying the LND epitope in PA, which was not present in the original crystal structure PDB 1ACC [38] but was later resolved in PDB 1TZN, when PA was bound to a domain of the anthrax toxin receptor CMG2 [35,39]. Second, work by Lim et al. demonstrated that neutralizing monoclonal Abs (mAbs) elicited through the immunization of mice with full-length LF, and which bound sequences in the α2-α3 helices of domain 3, were completely inhibited with linear peptides in this region [40]. Separately, work by Nguyen et al. also demonstrated that neutralizing mAbs elicited through the immunization of mice with LF were partially inhibited with linear peptides from the α2-α3 helices of domain 3 [41]. Together, these data were sufficiently compelling to suggest that linear neutralizing epitopes might be present in the α2-α3 helices of domain 3 of LF, but to date, there has been no evidence that neutralizing Ab could be elicited through immunization with peptide sequences from this region, nor from any peptidic region of full-length LF.

**Figure 2:**
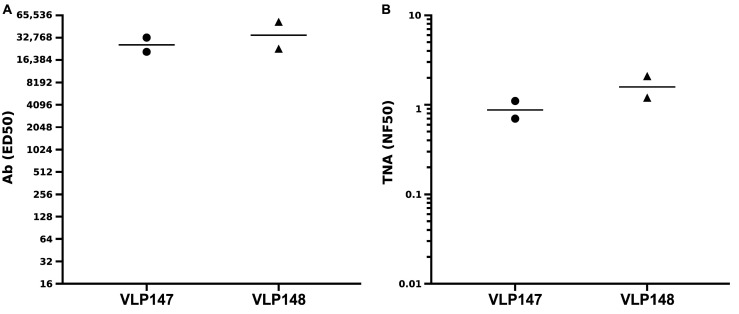
Immunogenicity of the LF-VLPs in rabbits. Antibody (**A**) and TNA (**B**) titers from week 8 sera (3 weeks post-booster immunization) from groups (*n* = 2) of NZW rabbits immunized two times with either the VLP147 or VLP148 nanoparticles in alum/MPLA. Each circle or triangle represents an individual rabbit data point, and the horizontal lines are geometric means.

We therefore proceeded to molecularly construct two VLPs displaying a longer (VLP147) and shorter (VLP148) version of putative linear neutralizing peptide sequences from LF which we hypothesized could contain neutralizing epitope(s) (Table 1 and Figure 1B,C). The decision to display the putative sequences on VLPs was related, in part, to the established immunogenicity of VLPs, whether derived from the human or woodchuck Hepatitis B capsid protein, when using human-use or even no adjuvants [36,42]. This was particularly important in light of our prior work with the LND peptide-containing immunogens, which, though highly protective in high-dose anthrax spore inhalation challenges in rabbits when incorporated into MAPs or a recombinant protein, nevertheless required five immunizations to attain significant levels of neutralization [25,26,27]. Following the molecular construction and purification of the VLPs and the confirmation of antigenicity by ELISA, the VLPs were used to immunize two groups of NZW rabbits (*n* = 2) at day 0 and week 5 with 300 μg of the respective VLPs using Alum/MPLA. When 8-week rabbit sera (3 weeks post-booster immunization) was analyzed in vitro, both VLPs displaying either the longer (VLP147) or shorter (VLP148) LF peptide sequences elicited high-titer Ab which bound full-length immobilized LF by ELISA (Figure 2A) and neutralized LeTx in vitro in the TNA (Figure 2B).

**Figure 3:**
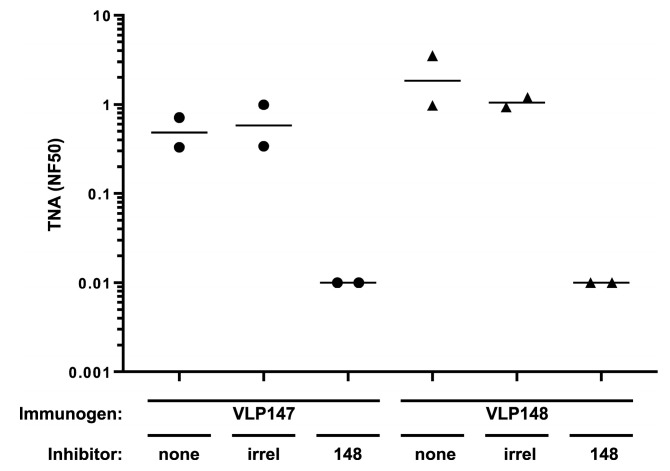
Mapping of the LF neutralization epitope to the VLP148 nanoparticle. Shown are TNA titers from 8-week sera (3 weeks post-booster immunization) from VLP147- and VLP148-immunized rabbits. Rabbit sera was pre-incubated with the indicated inhibitors followed by assessment in the TNA. Each circle or triangle represents an individual rabbit data point, and the horizontal lines are geometric means.

To assess whether the neutralizing antibody present in the sera from rabbits immunized with the VLP147 and VLP148 were eliciting Ab to the same neutralizing epitope, we performed an inhibition TNA, whereby sera from VLP147- and VLP148-immunized rabbits were pre-incubated with either media alone, an irrelevant VLP, or VLP148, prior to assessment in the TNA. As shown in Figure 3, pre-incubation with media alone or an irrelevant VLP had no significant effect on the neutralization levels for any of the VLP sera, while pre-incubation with the VLP148 completely inhibited the neutralization in the 8-week sera of all VLP147- and VLP148-immunized rabbits. The results confirmed that both VLPs share a common neutralizing motif contained within the sequences present in the shorter VLP148 nanoparticle.

### 2.2. Optimization and Characterization of VLP148

Having experimentally determined that VLP148 contained the neutralizing epitope, we molecularly constructed and purified two additional VLPs which contained small N- or C-terminal extensions of the VLP148 LF peptide sequence to test for enhanced immunogenicity in rabbits (Table 2).

**Figure 4:**
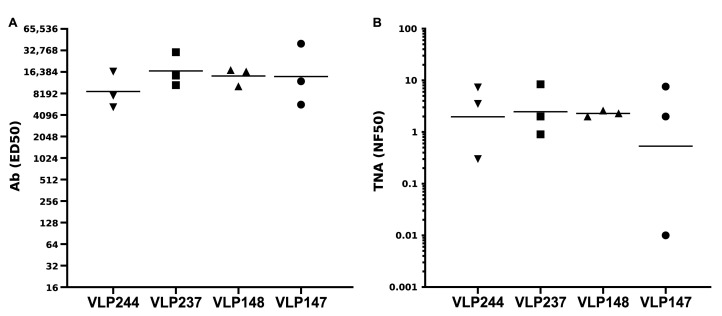
Comparative immunogenicity in rabbits of VLPs displaying modifications to the VLP148 sequence. Antibody (**A**) and TNA (**B**) titers from week 8 sera (3 weeks post-booster immunization) from groups (*n* = 3) of NZW rabbits immunized two times with the indicated VLP nanoparticles in alum/MPLA. The sequences for the respective VLPs are displayed in Table 2. There were no significant differences in either Ab or TNA titers. Each triangle, square or circle represents an individual rabbit data point, and the horizontal lines are geometric means.

The two resulting VLPs, VLP244 and VLP237, together with the VLP147 and VLP148, were then evaluated for immunogenicity in rabbits (*n* = 3) using the identical immunization protocol employed in the first rabbit immunogenicity study. As shown in Figure 4A, all four VLPs, formulated in Alum/MPLA, elicited high-titer Ab which was reactive with immobilized LF by ELISA. Importantly, all rabbits, with the exception of a single rabbit in the VLP147-immunized group, elicited neutralizing Ab (Figure 4B). There were no significant differences, however, in either the Ab or neutralization titers among the rabbit groups. Based on these results, the VLP148 became the lead candidate to move forward for further analysis.

**Figure 5:**
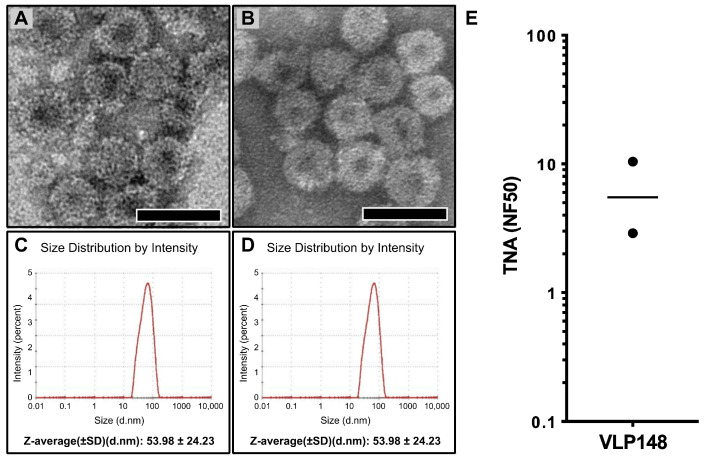
Transmission electron microscopy, dynamic light scattering, and immunogenicity of the lyophilized and reconstituted VLP148. Transmission electron microscopy of the non-lyophilized (**A**) and lyophilized/reconstituted VLP148 (**B**). Magnification = 40,000X Scale bar = 50 nm. In (**C**,**D**) are shown the results from dynamic light scattering sizing for the non-lyophilized (**C**) and lyophilized/reconstituted (**D**) VLP148 nanoparticle. Panel (**E**) displays the TNA titers (GMT = 5.50 NF50) from sera obtained at week 8 from a group of rabbits (*n* = 2) immunized twice (wk 0, wk 5) with the low-endotoxin, lyophilized/reconstituted VLP148 using the Alum/MPLA adjuvant. Each circle represents an individual data point, and horizontal line is the geometric mean.

Though our prior work demonstrated that the presence of endotoxin does not increase the immunogenicity of human hepatitis B VLPs in mice [43], and we have not seen any evidence for endotoxin increasing the immunogenicity of WHc VLPs in rabbits (unpublished), we evaluated whether the reduction of endotoxin in the purified VLP148 would reduce the immunogenicity in rabbits. We used the established Triton X-114 method to reduce the endotoxin levels in the purified VLP148 to less than 20 EU/mL. We then proceeded to lyophilize a fraction of the low-endotoxin VLP148 preparation and reconstituted the lyophilized material after approximately one week, during which time it was stored at 4 °C. The low-endotoxin, soluble (not lyophilized) fraction of the preparation and the low-endotoxin, lyophilized/reconstituted VLP148 fraction were then compared using transmission electron microscopy (TEM) and dynamic light scattering (DLS). As shown in Figure 5A (non-lyophilized) and Figure 5B (lyophilized/reconstituted) there were no discernible differences in the morphology of the nanoparticles (and Supplementary Figure S1). There were also no differences evident in the sizing of the VLPs by DLS regardless of whether they had remained in soluble form (Figure 5C) or had undergone lyophilization and reconstitution (Figure 5D) following purification. Lastly, we evaluated the low endotoxin, lyophilized/reconstituted VLP148 for immunogenicity in rabbits (*n* = 2) using our standard immunization protocol using the Alum/MPLA adjuvant. As shown in Figure 5E, neither the reduction of endotoxin nor the lyophilization/reconstitution appeared to diminish the elicitation of neutralizing Ab in 8-week rabbit sera (3 weeks post-boost).

### 2.3. Evaluation of the VLP148 with and Without the Protamine Domain

**Figure 6:**
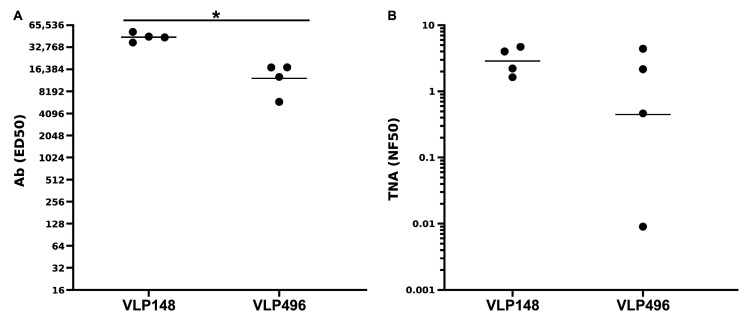
Comparative immunogenicity of the VLP148 and VLP496 in rabbits. Groups of rabbits (*n* = 4) were immunized at week 0 and week 5 with either the low-endotoxin, lyophilized/reconstituted VLP148 or the low-endotoxin, lyophilized/reconstituted VLP496, which lacks the C-terminal 40 amino acids containing the poly-arginine protamine domain. Ab (**A**) and neutralization (**B**) titers were assessed in 7-week sera, 2 weeks following the booster immunization. Ab titers in sera from the VLP148-immunized rabbits (GMT = 45,183) were significantly higher than the Ab titers in the sera from the VLP496-immunized rabbits (GMT = 12,339) (* *p* = 0.0002). Though the neutralization in the VLP148-immunized rabbits (GMT = 2.89 NF50) trended higher compared to the neutralization in the VLP496-immunized rabbits (GMT = 0.45 NF50), the results did not reach significance (*p* = 0.30). In both (**A**,**B**), Each circle represents an individual rabbit data point, and the horizontal lines are geometric means.

Both the human Hepatititis B and woodchuck Hepatititis B capsid proteins contain a C-terminal tail comprising a high density of repeating arginines, often referred to as the “protamine domain”. These poly-arginine tails are integral to encapsidation of bacterial RNA within the VLPs which serves to stimulate Toll-like receptor 7 and contributes to the immunogenicity of the VLP nanoparticles [37,43]. In some studies, for example, in human clinical trials of the human Hepatitis B VLPs targeting malaria, the protamine domain was replaced with C-terminal helper T-cell sequences from malaria [42]. To determine whether the protamine domain contributed to the immunogenicity of the VLP148 in rabbits, we molecularly constructed a version of the VLP148, designated VLP496, in which we replaced the C-terminal 40 amino acids with a C-terminal cysteine at a.a. 149. The VLP496 was purified, underwent endotoxin removal, and was lyophilized/reconstituted akin to the low-endotoxin, lyophilized/reconstituted preparation of the VLP148 described above. The two identically-prepared VLPs were then assessed for immunogenicity in rabbits (*n* = 4) using the standard protocol described above utilizing Alum/MPLA adjuvant. As shown in Figure 6A, though both VLPs elicited Ab in 7-week sera (2 weeks post-boost) which reacted with immobilized LF, VLP148, which contains the protamine domain, was significantly more immunogenic by ELISA (*p* = 0.0002). The assessment of the respective 7-week sera by TNA revealed a trend towards higher neutralization in the VLP148-immunized rabbit sera but it did not reach significance (*p* = 0.30).

## 3. Discussion

The currently licensed vaccine for anthrax in the U.S., Biothrax^®^ (formerly AVA), does not contain immunogenic levels of LF [44]. Conversely, the approved vaccine for anthrax in the UK, anthrax vaccine precipitated (AVP), is known to contain small amounts of LF which has been shown to contribute significantly to the elicitation of neutralizing immunity in vaccinees [45,46]. Our earlier work evaluating the elicitation of Ab in rabbits inoculated with AAV expressing either LF or PA63 demonstrated that LF can elicit high titer neutralization that is equivalent in magnitude to that detected in rabbits inoculated with AAV expressing PA [14]. Others have also demonstrated the efficacy of eliciting Ab to full-length domains of LF which have proven successful at neutralizing LeTx in vitro and mediating protection from LeTx challenge in mice, and partial protection from anthrax inhalation spore challenge in rabbits [47,48]. Additionally, a number of studies have identified mAbs elicited to full-length LF which are capable of LeTx neutralization in vitro, and protection from LeTx challenge and Sterne spore challenge in vivo [40,41,49]. Based on such data, in combination with crystal structure analysis, we hypothesized that a peptide region in the α2-α3 helices of domain 3 of *B. anthracis* lethal factor might represent a peptidic candidate for eliciting LF-specific neutralizing Ab. Our overall objective was to identify a linear LeTx-neutralizing epitope which could be displayed on the WHc VLP for the successful elicitation of neutralizing Ab, and to combine this VLP with an LND-specific vaccine as a prototype lyophilizable, epitope-specific, bivalent vaccine for anthrax. Our data demonstrate that the VLP148 nanoparticle consistently elicits protective levels of neutralizing Ab in rabbits following two immunizations using human-use adjuvants. To our knowledge, this is the first demonstration of the elicitation of LeTx-neutralizing Ab to an LF peptide sequence. Moreover, the data show that lyophilization of the VLP has no deleterious effect on the elicitation of humoral immune responses, nor does the reduction of endotoxin in the VLP preparation to very low levels. The currently licensed vaccine for anthrax, Biothrax^®^, and the licensed anthrax vaccine in the UK, AVP, both require a cold-chain, as the vaccines cannot be lyophilized with concomitant maintenance of immunogenicity. This, in turn, requires the replacement of existing vaccine stocks at regular intervals, and at significant cost, as soluble PA undergoes chemical alterations over time, including de-amidation, which is associated with a reduction in immunogenicity [20,21]. In general, the ability to lyophilize vaccines is also highly advantageous for the delivery of vaccines at their point-of-contact with vaccinees which at times may not be amenable to cold-chain requirements.

The presentation of the LF peptide sequences on the WHc VLP appears to be critically important for the strong immunogenicity of the nanoparticle while employing human-use adjuvants. This is consistent with our work and the work of others using both WHc and human Hepatitis B capsid protein VLPs in other models and relates in part to the antigen presentation of the nanoparticles to B cells, the abundance of ubiquitously present sources of T-cell help in these capsid proteins, and the high-density array of the target sequences on the VLP nanoparticle [36,37,42,50]. In addition, the presence of encapsidated bacterial RNA in the VLP nanoparticle, which has been shown to stimulate TLR-7, also likely contributes to the immunogenicity of the VLPs in mice and rabbits as supported by our work and the work of others [37,43]. Our prior studies with the LND peptides, while distinct from the LF peptides evaluated in the current work, nevertheless demonstrated the overall weak immunogenicity of peptide sequences displayed as MAPs when employing human-use adjuvants, including Alum/MPLA, despite ultimately eliciting protective immunity in rabbits from aerosol spore challenge with *B. anthracis* Ames strain [27].

Lacy et al. demonstrated that the N-terminal domains of both edema factor and lethal factor interact with PA to enable the binding and translocation of LF and EF into the cytosol. Using deletion mutagenesis, they determined that the critical amino acids for these interactions spanned from a.a. 40–263 of LF, which is N-terminal to the regions targeted by the LF-VLP [39]. We would hypothesize that antibodies elicited to the LF-VLP vaccine bind domain 3 in LF prior to the binding and translocation of LF into the cytosol, and likely inhibit the conversion of lethal factor to lethal toxin intracellularly through steric interactions, or interfere with LeTx substrate-recognition. This is consistent with the proposed mechanisms of neutralization for mAbs which bind in this region [41].

An LF-VLP could theoretically be combined with a vaccine displaying the LND epitope also displayed on the WHcAg-VLP (in press, Microorganisms). Antibody elicited to the LND epitope has previously been demonstrated to protect rabbits from high-dose Ames strain inhalation spore challenge when used alone [25,26,27]. The addition of a second neutralizing epitope in the form of an LF-VLP would be expected to not only eliminate the single-point-of-failure associated with a vaccine targeting only the LND, but would theoretically decrease the time to achieve the levels of neutralization associated with protective immunity as determined in the rabbit and NHP models of experimental inhalation anthrax [5]. The levels of neutralization elicited in vivo employing a vaccine displaying two distinct neutralizing epitopes would likely be additive based on our in vitro work, and the work of others using combinations of mAbs against PA and LF, though in vivo testing would be required to test this hypothesis with a bivalent vaccine [5,49]. Such a bivalent vaccine would also elicit neutralizing specificities which are non-overlapping with the neutralizing specificities elicited by Biothrax^®^ and other PA-based vaccines, and as such, could be assessed for additivity when used with such vaccines, and could potentially be found useful for individuals who respond poorly to PA-based vaccines [24]. Finally, a bivalent vaccine comprising the LND and LF epitopes could be strategic in the unlikely, but theoretically possible scenario of the malicious re-engineering of PA to circumvent some of the neutralizing specificities responsible for protective immunity with PA-based vaccines, a contingency for which proof-of-principle has been demonstrated [33,49,51].

## 4. Materials and Methods

### 4.1. Construction, Purification, and Characterization of Recombinant WHcAg LF-VLPs

VLPs were constructed based on the full length WHcAg (accession M18752) gene sequence codon-optimized for *E coli* expression inserted into a pUC19 vector in place of the multiple cloning site. The putative LF neutralizing epitopes were engineered to be encoded between amino acids 78 and 79 of the core protein gene [52]. WHcAg constructs were transformed into DH5alpha-competent *E. coli*, grown in Terrific Broth and lysed by passage through an EmulsiFlex-C3 (Avestin, Ottawa, ON, Canada). The lysate was clarified by centrifugation and the particles were selectively precipitated by the addition of solid ammonium sulfate to 35% saturation (208 g/L). Precipitated VLPs were redissolved in minimum buffer (10 mM of Tris, pH 8), and diafiltered with approximately 5 volume exchanges of final formulation buffer in a hollow fiber cartridge with a 750 K molecular weight cutoff (WaterSep BioSeparations, Marlborough, MA, USA). The purified VLPs were 0.2 μm sterile-filtered, characterized, and aliquoted. Characterization included confirmation of antigenicity by ELISA, agarose gel electrophoresis, SDS-PAGE, and TEM as described [52,53]. The size distribution of the VLPs were determined by DLS on a Zetasizer Nano ZS instrument (Malvern, UK) [54]. For some immunization studies, the VLPs were subjected to endotoxin removal using extraction with Triton-X114 (Sigma, St Louis, MO, USA) and endotoxin levels were determined using the limulus amebocyte assay (Thermofisher, Pittsburgh, PA, USA) to be less than 20 EU/mL. For some studies, the purified VLPs were lyophilized, stored at 4 °C, and reconstituted in molecular biology-grade water prior to use.

### 4.2. Animals and Vaccinations

For the rabbit studies, male and female New Zealand white rabbits (Covance Research Products, Denver, PA, USA) weighing approximately 2.5 kg were primed on day 0 using a combined s.c. and i.m. route with 300 μg of a VLP mixed 1:1 (vol:vol) with Alhydrogel (InvivoGen, San Diego, CA, USA) containing 100 μg of monophosphoryl lipid A (MPLA, Avanti Polar Lipids, Birmingham, AL, USA). For most experiments, except where noted, rabbits were boosted at 5 weeks post-priming immunization with 300 μg of the respective VLP mixed with the same Alum/MPLA adjuvant combination. For procurement of antisera, small volume bleeds (<3.0 mL) were obtained from rabbits at varying time points as outlined in the figures and figure legends. Rabbit experiments were designed to minimize the number of animals used to establish proof-of-concept, or alternatively, were powered to assess significance. All rabbits were cared for in accordance with the standards of the Association for Assessment and Accreditation of Laboratory Animal Care, and all protocols were approved by the Institutional Animal Care and Use Committee of Covance Research Products (Denver, PA, USA; IACUC approval 19 March 2019, protocol #A191114).

### 4.3. Enzyme-Linked Immunosorbent Assay

Antibody responses were assessed by ELISA as described previously [35]. Briefly, Ab titers were determined following serial two-fold dilutions of serum and represent the reciprocal dilution at the EC50 which was established using nonlinear regression to fit a variable slope sigmoidal equation to the serial dilution data. All analysis was performed using Prism 10.0 (GraphPad Software, Inc., San Diego, CA, USA).

### 4.4. Toxin Neutralization Assay

The ability of antibody to block LeTx cytotoxicity in vitro was assessed in the TNA using the RAW264.7 cell line (American Type Culture Collection, Manassas, VA, USA) as described [55]. This assay measures whether Ab, antisera, or inhibitors can be pre-incubated with LeTx to prevent the cytotoxicity of the LeTx on the susceptible RAW264.7 macrophage target cell line. For neutralization studies, approximately 120 ng/mL of PA (PA 171E, List Labs, Campbell, CA, USA) and 40 ng of LF (LF169L, List Labs, Campbell, CA, USA) were used in the TNA. Contemporaneous toxin dose 50% (TD50) were separately determined for PA and LF with each TNA to validate that the toxin concentrations used in each TNA were sufficient for greater than 90% cytotoxicity (4 TD50s), and irrelevant serum samples were assessed in each TNA to rule out non-specific Ab or serum effects on neutralization. The reciprocal of the effective dilution (ED) protecting 50% of the cells from cytotoxicity (ED50) [56] was determined for each serum by using nonlinear regression to fit a variable-slope sigmoidal equation to the serial dilution data set using Prism 10.0. TNA results were displayed as NF50s (NF50 = ED50 sample/ED50 AVR801) which normalizes the TNA results to AVR801 (Human anti-AVA reference serum AVR801, Lot 2, BEI Resources, Manassas, VA, USA) [5]. The standard TNA has a lower limit of quantification of 16. Samples with TNA results below this limit were assigned a value of 8 (NF50 = 0.01). Horizontal lines represent geometric mean titers (GMT). For inhibition TNAs, sera were pre-incubated with media alone or 20 μM of inhibitor VLPs as outlined in the figure legend for 30 min at room temperature, prior to the assessment of the samples in the TNA.

### 4.5. Statistical Analysis

Due to non-Gaussian distribution of data, Mann–Whitney and Kruskal–Wallis tests were used for comparing titers between two groups or more than two groups, respectively. For all statistical analysis, a *p* value of <0.05 was considered significant. All statistical analyses were performed using GraphPad Prism version 10 (GraphPad Software, San Diego, CA, USA).

## Figures and Tables

**Table 1:** Amino acid sequences displayed by preliminary LF-VLPs. The amino acid sequence numbers are consistent with PDB 1J7N.

VLP Construct	Amino Acids	Sequence
VLP147	332-366	TEEKEFLKKLQIDIRDSLSEEEKELLNRIQVDSSN
VLP148	342-361	QIDIRDSLSEEEKELLNRIQ

**Table 2:** Amino acid sequences displayed by modified LF-VLPs. The amino acid sequence numbers are consistent with PDB 1J7N.

VLP Construct	Amino Acids	Sequence
VLP147	332-366	TEEKEFLKKLQIDIRDSLSEEEKELLNRIQVDSSN
VLP148	342-361	QIDIRDSLSEEEKELLNRIQ
VLP237	342-366	QIDIRDSLSEEEKELLNRIQVDSSN
VLP244	338-366	LKKLQIDIRDSLSEEEKELLNRIQ

## Data Availability

The original data presented in this study are fully included in the article and the Supplementary Materials.

## Supplementary Materials

The following supporting information are available online at https://www.mdpi.com/article/10.3390/toxins17080422/s1: Figure S1: Fully uncropped fields for TEMs as captured for Figure 5A,B.

## Abbreviations

The following abbreviations are used in this manuscript:

VLP	Virus-like particle
TNA	Toxin neutralization assay
LF	Lethal Factor
MAP	Multiple antigenic peptide
LeTx	Lethal toxin
WHcAg	Woodchuck Hepatitis core antigen
