# Supplementary material for: A Lyophilizable Nanoparticle Vaccine Specific for a Novel Linear Neutralizing Epitope in the α2-α3 Helices of Domain 3 of Lethal Factor from Bacillus anthracis
Source: Toxins (Basel). 2025 Aug 20;17(8):422. doi: 10.3390/toxins17080422 (PMC12389915; doi:10.3390/toxins17080422)
Supplement: Supplementary file 1 [file toxins-17-00422-s001.zip › toxins-3803758-supplementary.pdf]

A Lyophilizable Nanoparticle Vaccine Specific for a Novel Linear Neutralizing Epitope in the α2-α3 Helices of Domain 3 of Lethal Factor from *Bacillus anthracis*

Jon Oscherwitz, Kemp Cease, David Milich, Thomas Braun, Fen Yu and David Whitacre

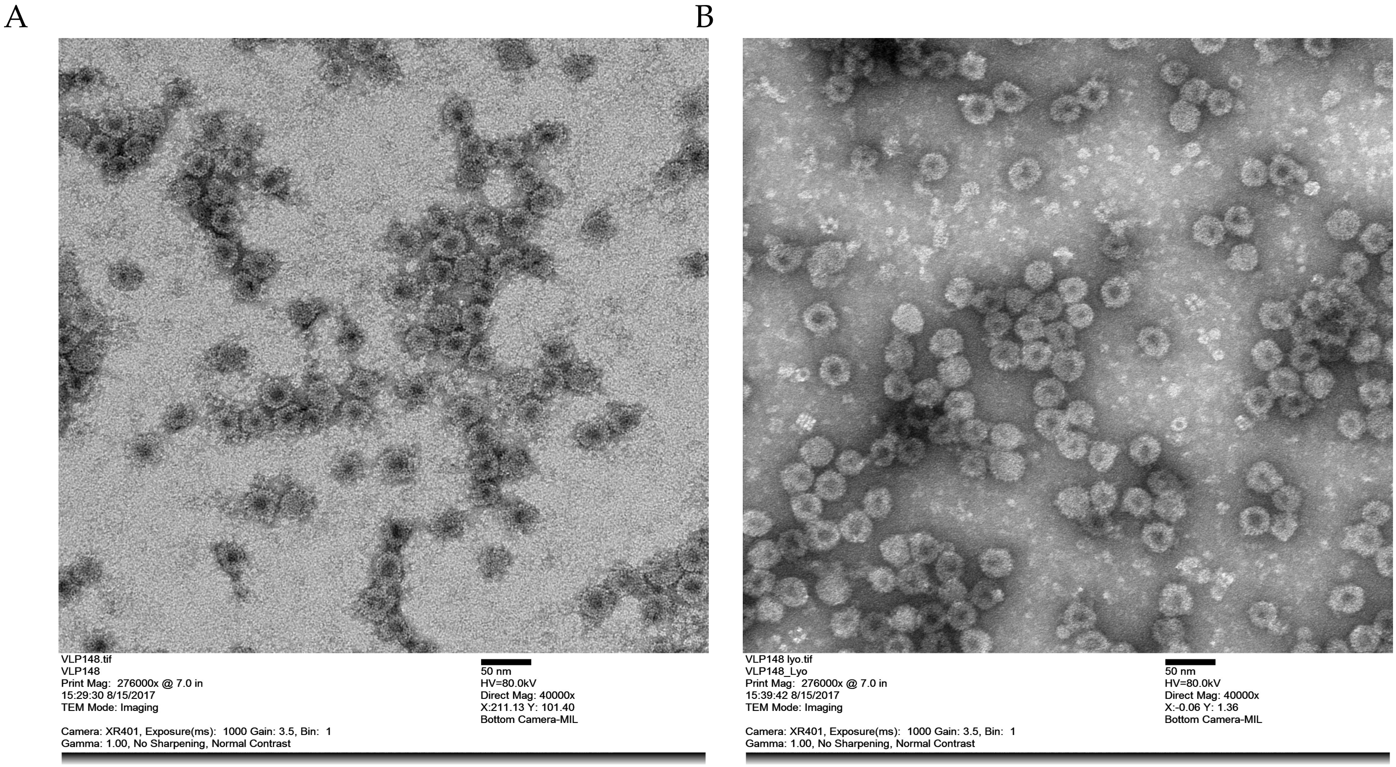

Figure S1. Uncropped transmission electron micrographs (TEM) from Figure 5. A. Purified VLP148 before lyophilization. B. VLP148 after lyophilization and reconstitution. Details from the transmission microscope, including scale bar, are at the bottom of each uncropped image.
